# Smart Wound Dressings for Diabetic Chronic Wounds

**DOI:** 10.3390/bioengineering5030051

**Published:** 2018-06-26

**Authors:** Elizabeth Gianino, Craig Miller, Jordon Gilmore

**Affiliations:** Bioengineering Department, Clemson University, Clemson, SC 29632, USA; egianin@g.clemson.edu (E.G.); clm6@g.clemson.edu (C.M.)

**Keywords:** diabetes, chronic wounds, smart wound dressing, biochemical sensor

## Abstract

Given their severity and non-healing nature, diabetic chronic wounds are a significant concern to the 30.3 million Americans diagnosed with diabetes mellitus (2015). Peripheral arterial diseases, neuropathy, and infection contribute to the development of these wounds, which lead to an increased incidence of lower extremity amputations. Early recognition, debridement, offloading, and controlling infection are imperative for timely treatment. However, wound characterization and treatment are highly subjective and based largely on the experience of the treating clinician. Many wound dressings have been designed to address particular clinical presentations, but a prescriptive method is lacking for identifying the particular state of chronic, non-healing wounds. The authors suggest that recent developments in wound dressings and biosensing may allow for the quantitative, real-time representation of the wound environment, including exudate levels, pathogen concentrations, and tissue regeneration. Development of such sensing capability could enable more strategic, personalized care at the onset of ulceration and limit the infection leading to amputation. This review presents an overview of the pathophysiology of diabetic chronic wounds, a brief summary of biomaterial wound dressing treatment options, and biosensor development for biomarker sensing in the wound environment.

## 1. Background

Diabetes mellitus, an increasing health concern that affects more than 9% of the population over the age 18, is the seventh leading cause of death in North America [1,2]. A number of severe health concerns are associated with diabetes, such as peripheral arterial disease, neuropathy, limited joint mobility, abnormal foot pressures, minor trauma, and foot deformity. Improved treatment of diabetes could significantly decrease associated healthcare costs, given that the cost of healing a single ulcer, infected ulcer, and amputation are estimated at $8000, $17,000, and $45,000, respectively [3]. Patients who suffer from diabetes have a 15–25% chance of developing a chronic wound. Chronic wounds associated with diabetes include foot, venous and pressure ulcers [4]. Diabetic foot ulcers (DFUs) are classified as chronic, non-healing wounds that create a disruption in the skin with a frustrated and extended healing process. With a global prevalence of 6.3%, DFUs place a heavy burden on public health [5]. Peripheral neuropathy is one of the most frequent precursors of diabetic ulceration and is manifested when the peripheral nerves in the limbs become damaged. The loss of sensation impairs the ability to sense excessive pressure or pain from minor injuries. The lack of response to what may initially be minor incidents, combined with the poor blood circulation and impaired healing capacity of diabetic patients eventually leads to ulceration [6]. Those affected by DFUs and other chronic wounds are at increased risk for lower extremity amputation due to the threat of osteomyelitis and/or sepsis resulting from wound infection. Several studies have concluded that 85% of amputations are preceded by ulcers [7], and the incidence of new ulcer formation at a collateral wound site may be up to 50% [8].

## 2. Chronic Inflammation in Diabetic Wounds

The complete pathophysiology of ulceration is still unclear; however, clinicians and wound care specialists widely assert that delayed healing is due to complications of peripheral arterial diseases, neuropathy, inflammation, and ischemia [9]. A combination of impaired growth factor production, angiogenic response, collagen accumulation, fibrosis, and abnormal pressure may result in ulceration and chronic cracks in the feet. Ultimately, the wound healing response at the ulceration site can be characterized by symptoms of a frustrated and prolonged inflammatory response. 

Inflammation becomes activated within a day of ulcer formation and can last up to two weeks or longer. Inflammatory cells, such as neutrophils, macrophages, T-lymphocytes, fibroblasts, and prostaglandin E2 (PGE2) secrete enzymes that result in pain, redness, warmth, and swelling necessary for the healing cascade [10,11].

Neutrophils activate upon responding to chemotactic signals that allow for cell localization in the infected area. In a process called margination, neutrophils roll about the vasculature and migrate into the wound site with the help of cell adhesion molecules (CAMs) through diapedesis [11]. The lack of functional adhesion molecules at this point in the inflammatory response delays healing [11]. Neutrophils release elastase and collagenase for the purpose of destroying and removing damaged structural proteins. They also produce tumor necrosis factor alpha (TNF-α) and interleukins 1 (IL-1), to allow for fibroblast and epithelial cell homing [11].

Macrophages are the second key inflammatory cells needed for phagocytosis and releasing cytokines and growth factors, such as platelet derived growth factors (PDGF), transforming growth factors beta (TGF-β), beta fibroblast growth factors (β-FGF), TNF-α, IL-1, and IL-6 for promoting fibroblast proliferation and reepithelialization. Finally, lymphocytes, enter the wound and produce IL-2, needed to aid in fibroblast recruitment [11].

The role of fibroblasts in the healing process is significant because they produce matrix metalloproteinases (MMPs), which destroy impaired structural protein. They also release proteins that provide structural support for the new extracellular matrix [11]. In chronic wounds, fibroblasts are non-receptive to cytokines and growth factors; as a result, their activity is impaired and there remains a distorted extracellular matrix [11]. Fibroblasts release tissue inhibitor metalloproteinases (TIMPs) in order to regulate the effects of MMPs. Unregulated MMPs are overwhelming and destroy the old ECM in addition to fresh structural proteins [11].

Prostaglandin E2 (PGE2) is a hormone that is produced by blood vessels and promotes vasodilation and angiogenesis by inducing vascular endothelial growth factors (VEGFs). Thus, PGE2 is a vasodilator needed to prevent hypoxia. Individuals with DFUs suffer from vascular disease and peripheral arterial disease due to lower levels of PGE2 [10].

Primarily, ulcers occur through minor trauma in the presence of sensory neuropathy. Often, neuropathy is undiagnosed until ulcer formation or pain develops. Diabetes is associated with nerve damage, which results from diseased vasa nervorum and interacting metabolic abnormalities [12]. Hyperglycemia may inhibit the production of nitric oxide, ultimately creating an environment that is more susceptible to reactive oxygen species, such as superoxide and hydrogen peroxide. This can disrupt the productivity of endothelium-derived vasodilator and can lead to a cascade of platelet aggregation, inflammation, thrombosis formation, and atherosclerosis of small vessels neighboring peripheral nerves, eventually leading to peripheral neuropathy. Although damage from neuropathy is irreversible, controlling blood glucose levels can prevent further ulceration. Other behavioral changes such as monitoring hyperglycemia, hypertension, smoking, cholesterol levels, and heavy alcohol use can prevent injury while promoting a better healing environment.

Patients with diabetes may also have vascular disease and ischemia, which can contribute to delayed healing. In fact, ischemia is a major contributor in 90% of DFU patients who undergo amputation [13]. Therefore, there is a high correlation between ischemia and atherosclerosis and highly infected DFUs. Capillary thickening can both interfere with normal flow of inflammatory cells and also create inelasticity, ultimately making it difficult for vasodilation needed for responding to local injury [13].

Because DFUs are chronic, treating infection can be challenging. Once an ulcer forms, infection can build and spread due to open air wounds, lack of sterility and a loss of innate barrier function [13]. Dysfunctional leukocytes are common among the inflammatory response of patients with diabetes [14]. Additionally, phagocytosis is reduced due to hyperglycemia, fibroblast migration is hindered, and the protective barrier and repair mechanism slows down. Normal wound exudate levels are optimized for healing, while in chronic wounds, exudate levels contain higher concentrations of MMP [11]. MMP-2 and MMP-9 protein concentrations were 10 times and 25 times higher in pressure ulcer fluids than surgical fluids, respectively [15]. The majority of pathogens found in DFUs are *Staphylococcus aureus*, *Enterococcus faecalis*, *Pseudomonas aeruginosa*, and anaerobic bacteria [4]. Pathogen concentration levels above or equal to 10^5^ Colony Forming Units (CFU) per gram are capable of interfering with the wound healing process [4]. Bacteria proliferate toward this critical concentration level, and a biofilm forms as bacteria encase themselves within extracellular matrix substances of polysaccharides and lipids. Resistance to immunological responses becomes a significant problem for patients with infected chronic wounds.

## 3. Current Treatment and Challenges

DFUs can be diagnosed into four different depth ischemic classifications. Table 1 displays the major classification methods and their respective stages for evaluation of ulceration. Clinicians responsible for diagnosis may subscribe to any of the classification methods listed below or none of them, making treatment decisions increasingly subjective in nature, and highly based on the level of clinician experience.

Once a chronic wound is properly categorized, management and treatment can start. Historically, earlier detection and diagnosis have yielded higher rates of healing [13]. If infection is present, the first priority of treatment is to stop progression to severe osteomyelitis or sepsis. Antibiotics for treating both the ulcer bed and osteomyelitis should be chosen based on the spectrum of infecting organisms. Infections typically contain a combination of *S*. *aureus* and *Escherichia coli*, which can be killed by applying aminopenicillin and penicillinase inhibitor as well as quinolone, metronidazole or clindamycin [19,20]. Other studies have indicated that intravenous options, such as imipenem, gentamicin, vancomycin, teicoplanin, rifampicin, or lenozoid can be effective [19,21]. Silver nanoparticles and many other hard metals, such as zinc, copper, and arsenic also contain antimicrobial properties, but must be carefully monitored for the development of a metal toxicity [22,23]. Revascularization can be made possible through angioplasty, thrombolysis, and most commonly, bypass surgery [6]. Off-loading is important since pressure beneath the ulcer bed can significantly increase build over time, leading to subsequent ulceration. Custom-made orthotic devices can assist in lowering plantar pressure. However, removable orthotics are only as helpful as a patient’s willingness to comply with clinician instructions.

Ulcers heal more quickly if the surface is clean [24]; physicians must debride impediments to healing, such as necrotic tissue and bacteria. Dressings can provide a warm, moist environment required for healing after debridement [25]. Some engineered dressings include hydrogels, hydrocolloid films, foams, alginates, etc. [26,27,28]. Common problems associated with some of these dressings have been dehydrating the ulcer bed, saturation with exudate, and/or the failure to properly apply antibiotics and growth factors needed to promote angiogenesis and granulation tissue.

Currently, there are a wide variety of commercially available polymeric wound dressings that have proven to enhance healing. Since treatment strategies depend on a unique combination of comfort, promotion of reepithelialization, prevention of further trauma, moisture, exudate wicking, antimicrobial properties, etc., the variance of these dressings are extreme.

Wound contact materials are placed over the wound in the evidence of minor exudation. Non-medicated dressings include paraffin gauze, while medicated include Xeroform^®^ (Covidien, Dublin, Ireland) [29]. Hydrogel, foam, and other absorbent dressings are primarily used in managing highly exudated wounds. However, hydrogels can also rehydrate a wound if moisture level is too low. Common absorbent dressings include Primapore^®^ (Smith & Nephew, London/Hull, UK), Mepore^®^ (Mölnlycke, Gothenburg, Sweden) and absorbent cotton gauze (BP 1988). Hydrogel dressings include ActiformCool^®^ (Activa) and Aquaflo^®^ (Covidien, Dublin, Ireland) [29]. Promgran Prisma^®^ (Systagenix) and Dermol/Ag™ (DermaRite Industries, North Bergen, NJ, USA) are two collagen matrix dressings that can transform into a biodegradable gel if exudate levels remain high within the wound [30]. Aquacel Hydrofiber^®^ (ConvaTec, Reading, UK) creates a soft gel-like material as it absorbs wound fluid, while maintaining a moist environment. Meliplex Ag (Molnlych Health Care, Gothenburg, Sweden) is a vapor-permeable, waterproof film, that regulates wound moisture while protecting the environment from bacterial invasion. Tegaderm™ (3 M Health Care) is another popular dressing that not only absorbs exudate, but is comfortable and easy to remove from fragile and sensitive tissue [30].

Film dressings are important to consider when a membrane layer is needed to allow for the passage of oxygen and vapor, while preventing the invasion of water, exudate, or bacteria [29]. Many polyurethane materials can be used for this purpose and is mentioned in Section 4.2.2. GranuDerm™ and Sentry™ (Acute Care Sollutions, LLC, Canton, OH, USA) are breathable films that rid the wound of water, dirt, and microbes, while prohibiting leakage. Similarly, Silverlon^®^ (Argentum Medical, LLC, Geneva, IL, USA) permits the passage of exudate, while preventing microbial invasion and contamination [30].

As mentioned before, silver has been used frequently to treat infected wounds. Allevyn (Smith & Nephew, London/Hull, UK) is a polyurethane film combined with foam containing silver sulphadiazine [26]. The release of antibacterial action was observed to last approximately 7 days. Dermacol/Ag™ (DermaRite Industries, North Bergen, NJ, USA) is a collagen matrix wound dressing that contains silver chloride in order to prevent bacterial colonization. Many of the foam or hydrogel dressings such as Promgran Prisma^®^ (Systagenix, Skipton, UK), Meliplex Ag (Molnlych Health Care, Gothenburg, Sweden), and Aqucel Hydrofiber^®^ (ConvaTec, Reading, UK) contain silver within their matrix to provide a protective barrier for not only fighting infection, but also allowing for an optimal healing environment [30].

Many of the commercially available wound dressings have shown potential for healing chronic DFUs, however, they lack a complete holistic approach. Wound dressings vary on the level of absorbency. One wet, absorbent dressing might be only practical for highly exudated wounds. On the other hand, a type of occlusive film might only be advantageous for wounds that need to maintain moisture [29]. A combination of layers of different material types needs to be developed to potentially fit the needs of all varieties of DFUs.

The treatment of chronic diabetic wounds requires the proper balance between experience-based intuition and science [31]. Modern management of ulcers is inefficient because preliminary assessment and diagnosis are often subjectively performed by clinicians. There is a need for an integral system that combines the use of the therapeutic components such as wound dressings and antibiotics with diagnostic components such as quantitative sensors to create a holistic treatment strategy for diabetic chronic wounds. Not only will the chances of wound healing rise, but also, the risk of new ulcer formation will decrease. Major developments in treatment strategies are needed for wound dressing design and quantitative diagnosis in order to decrease subjectivity and improve patient compliance. The subsequent sections highlight the state of the art in wound dressings, sensors, and composite smart wound dressings for the treatment of chronic diabetic wounds.

## 4. Current Wound Dressings

Wound dressings are needed to provide a barrier between the ulcer and the external environment. DFUs excrete wound fluids and have a prolonged healing process. Thus, the quintessential dressing is made of an antimicrobial material, maintains a moist environment, is permeable to oxygen, removes wound exudate, and allows the release of needed growth factors or drugs for the wound to facilitate proper proliferation and tissue remodeling [32]. There are myriad dressings for use in ulcer and chronic wound care, and the type of dressing used depends on the physiological parameters of the ulcer. Combinations of natural and synthetic polymers give rise to a more ideal material fit to not only heal the ulcer bed, but also to provide a foundation for reepithelialization.

While orthotics and skin grafts can play a crucial role in removing excess pressure and regenerating skin, wound dressings play a significant role in providing not only protection but also a factor for promoting natural healing. Recently, dressings can facilitate healing through additives that allow for a moist environment, removal of exudate, antibacterial effects, and the stimulation and proliferation of fibroblasts and keratinocytes at the site of injury [25,33]. None of these factors can be applied without patient compliance to facilitate application and removal without aggravating the symptoms [34]. A holistic approach should also consider minimizing cost. Thus far, no single dressing has met all the needs for proper wound healing, and therefore, further research is needed to explore a unique combination. The following section focuses on recent progress in natural and synthetic polymer-based wound dressings (Table 2) and clinical outcomes and the need to improve micro- and macro-scale geometry and overall architecture.

### 4.1. Natural Polymers

Natural polymers have been popular in research and clinical settings because their properties are biocompatible and closely related to the extracellular matrix. They mimic many biological systems to prevent the immunologic reactions caused by many synthetic polymers [35]. Additionally, natural polymers can be synthesized into polysaccharides, proteins, and polyesters by living organisms, or more recently, by fermentation of microorganisms [35].

#### 4.1.1. Cellulose

Cellulose is the most abundant organic polymer on Earth. It has been used in several wound healing applications since it releases phosphodiesterase growth factor, epidermal growth factor (EGF) and basic fibroblast growth factor (bFGF), all of which stimulate fibroblast growth and anti-inflammatory effects [36]. Beneficial properties of specifically bacterial cellulose include: hydrophilic surfaces, water uptake capacity, permeability, and tensile strength, all of which are comparable to the fibrous structure of collagen. Brassolatti et al., designed a bacterial cellulose dressing on third degree burn wounds in rats and noticed optimized healing response. Their results indicated that the use of bacterial cellulose dressings with and without lidocaine had comparable advanced repair outcomes, with both being more effective than the untreated control group. Skin appendages, mild inflammatory cell influx, collagen fiber organization and mild immunostaining were observed [37].

#### 4.1.2. Chitosan

Chitosan, a copolymer derived from chitin, is prominent in the exoskeletons of arthropods and the cell wall of fungi [38,39]. It can be fabricated to form a gelatin or film-like material, and its versatile effects promote strong adhesion to wound beds. Chitosan is a widely popular wound dressing material due to its multifunctional properties such as nonantigenicity, inertness, nontoxicity, biodegradability, biocompatibility, bioadhesiveness, antimicrobial properties, and hemostatic effects [40,41,42]. A chitosan based Opticell dressing (Medline Industries, Chicago, IL, USA) has hemostatic efficacy. Stricker-Kongrad et al., examined the Opticell dressing on heparinized rats with excisional wounds that mimicked debridement. Researchers noted that after removing the dressings, the total blood loss was significantly less than that of a typical gauze dressing. This indicated that Opticell dressings with chitosan have hemostatic effects that could be used to control bleeding associated with wound debridement [43]. Unlike cellulose, chitosan has beneficial antimicrobial effects. When crossed with cellulose, the composite in the form of film or hydrogel inhibited the growth of *E. coli* and *S. aureus*, while enhancing wound repair and epithelial regeneration in wound and burn infections [44,45].

#### 4.1.3. Collagen and Gelatin

**Figure 1:**
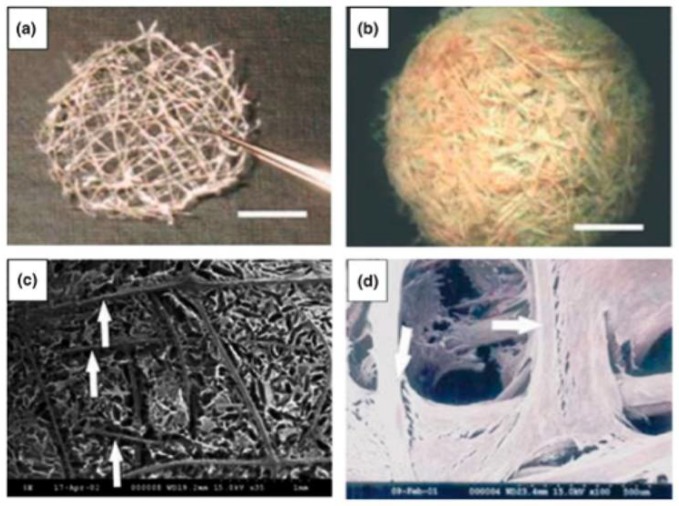
Collagen thread mesh imaged using optical microscopy (**a**); reinforced collagen sponge with collagen mesh (**b**); Collagen sponge reinforced by collagen mesh using SEM micrographs (**c**); and the incorporation of fibroblasts growing within collagen mesh using SEM micrographs (**d**). Reprinted by permission from Springer Nature: Biotechnology and Bioprocess Engineering reference [50]. Copyright 2008.

Another abundant natural polymer found in the ECM is collagen. Collagen is present in most epithelial and connective tissues, such as bone, cartilage, ligaments, tendon, and skin. It provides strength and integrity and is an essential component in cell-cell interactions that regulate anchorage, migration, proliferation, differentiation, and survival [22,46,47]. Collagen has been a key ingredient in promoting tissue granulation and angiogenesis and in inhibiting bacterial growth in chronic wounds. Collagen has been used as a carrier of reactive oxygen species, growth factors, and antibiotics. Wiegand et al., fabricated a collagen and cellulose composite that demonstrated a reduction of cytokines and proteolytic enzymes, indicating less inflammation in the wound bed [48]. A similar study assessed the effect of collagen dressings on the size, granulation tissue, and bacterial inhibition of chronic wounds. Patients with collagen-based dressings could avoid skin grafting since the presence of granulation tissue was a suitable alternative [49]. Figure 1 depicts a type of collagen mesh and the generation of fibroblasts around the scaffold.

Additionally, the dressing provided comfort, essential for patient compliance, as opposed to other conventional methods. Gelatin is a collagen derivative and is applicable to wounds that need a more hydrogel-like material [38]. It is biocompatible and degradable in physiological medium and can mimic many of the characteristics of the dermis. A research group that used bFGF-impregnated gelatin microspheres found that gelatin stimulates angiogenesis and fibroblast proliferation [51].

#### 4.1.4. Hyaluronic Acid

Hyaluronic acid (HA) presents a wide range of natural healing properties such as tissue integrity, lubrication, and water absorption. Similar to collagen, HA promotes mesenchymal and epithelial cell migration and differentiation, ultimately improving collagen deposition and angiogenesis for repair [38]. HA’s properties are sensitive to molecular weight, however. Campo et al., suggested that only medium molecular weight HA (MMWHA) could enhance wound repair; lower molecular weights contribute to further inflammation, and higher molecular weights might inhibit nutrient supply for tissue regeneration by blocking capillary formation [52,53]. Despite the sensitive functional aspect of HA, there have been promising results in the treatment of lesions where the loss of ECM was analyzed. Simman and colleagues noticed that during a clinical case series involving 12 patients with serious surgical wounds treated with HA-type wound dressings, all wounds developed granulation tissue [54].

### 4.2. Synthetic Polymers

Synthetic polymers are commercially available to overcome some of the limitations associated with natural polymers, such as inconsistent and non-reproducible chemical and physical composition. Synthetic polymers can be fabricated into various shapes, allowing for versatile function. Unlike natural polymers, most synthetics are insensitive to enzymatic and biological degradation and therefore have relatively more stable properties [55].

#### 4.2.1. Poly(lactide-co-glycolide)

Poly(lactide-co-glycolide) (PLGA) is a copolymer of polylactic acid (PLA) and polyglycolic acid (PGA). It is a degradable and biocompatible polymer, and its clinical use in drug delivery, suture applications in humans has been approved by the FDA. Although PLGA has yet to be approved for wound healing applications, significant research has been performed due to its efficacy in healing [56]. It exhibits mechanical strength and can conform into various shapes allowing for a plethora of processing types [57]. The ratio of lactide to glycolide units can greatly affect the release of bioactive substances and other pharmaceuticals. The larger the lactide units, the longer the polymer lasts before degrading [58]. Zheng et al., demonstrated that a PLGA and cellulose nanocrystal nanofiber membrane not only showed advantageous cytocompatibility, but also stimulated fibroblast adhesion, spreading, and proliferation. The release of neurotensin (NT), an inflammatory moderator, was also observed and researchers noted that the composite membrane allowed for sustained delivery of NT, ultimately promoting reepithelialization for the treatment of DFUs [59]. Another study created PLGA microspheres with high encapsulation of recombinant human epidermal growth factor (rhEGF) by solvent-evaporation. This research team observed an optimal growth rate of fibroblasts in and around the wound bed. Thus, the PLGA microsphere provided a compatible environment for the repair process as well as an optimal delivery route of rhEGF [60].

#### 4.2.2. Polyurethanes

**Figure 2:**
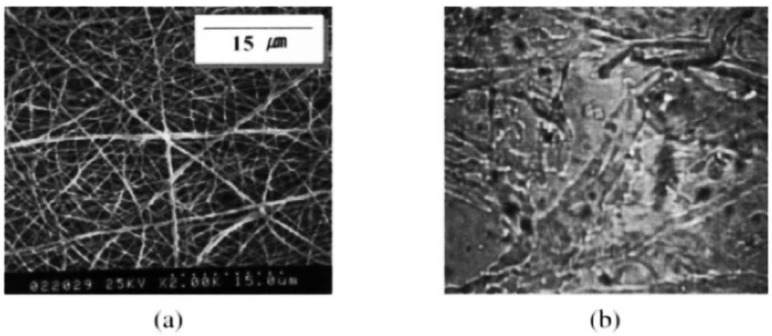
SEM image of PU electrospun fibers (**a**) and the Tegaderm™ wound dressing (**b**). Reprinted by permission from John Wiley and Sons: Journal of Biomedical Materials Research reference [61] Copyright 2003.

Polyurethanes (PU) possess not only a delivery route for growth factors and antibiotics, but also create a barrier that prevents bacteria from entering and further infecting the wound bed. PU is a suitable alternative synthetic polymer that provides a semi-permeable membrane. It imparts a moist environment and delivers bioactive substances for fighting infection and repair while protecting the wound from bacterial entry [57]. Additionally, PU-based nanofibers contain fluid drainage properties that decrease the risk of swelling, increased exudate, and wound desiccation [61]. Varma et al., proposed that a PU wound dressing could incorporate a combination of antibiotics, pain relievers, and protease inhibitors. While releasing these bioactive substances, the barrier layer can degrade in contact with the wound. Many times, PUs are used as the absorbent material, sandwiched between a contact layer and a waterproof film. Meliplex Ag (Molnlycke Health Care, Gothenburg, Sweden) is an example tri-layered wound dressing, and it has shown effective healing of many types of ulcers and burns [62]. Additionally, PU was electrospun to form a porous membrane that could wick away fluid from the wound, while preventing fluid buildup and wound desiccation. Figure 2 depicts electrospun PU fibers and a wound dressing prepared Tegaderm™, made out of a thin layer of PU and acrylic adhesives.

#### 4.2.3. Polyethylene Glycol

Polyethylene glycol (PEG) is another synthetic polymer desirable for wound dressings. It is a unique material, possessing hydrophilic, flexible, and compatible qualities. Often it can be blended with PLGA or chitosan for increased mechanical stiffness and stability [57]. It can also be used as a surface modifier for composite wound dressings allowing for better grip in the contact layer. Lee et al., fabricated a tri-bloc polymer of PLGA-PEG-PLGA and observed wound healing of DFUs in mice. Their results showed improved epithelium migration and collagen deposition, in addition to a higher wound closure rate [63]. A similar study used varying amounts of PEG interspersed with PLGA nanoparticles used to deliver recombinant human insulin as a potential wound healing agent. Researchers concluded that the sustained insulin delivery for 6 days by the PEG/PLGA vehicle led to enhanced cell proliferation [64]. Growth factors have a high affinity for PEG, and can be easily manipulated for local delivery [65]. Huang et al., analyzed the PEGylation of recombinant human acid fibroblast growth factor (rhaFGF) on diabetic wound healing. Higher expression of keratinocyte-specific genes was observed, and the rate of wound healing significantly increased [66].

#### 4.2.4. Polycaprolactone

Polycaprolactone (PCL), a biocompatible, biodegradable polymer that is resistant to many solvents, is similar to PLGA and it can be fabricated into many shapes and forms allowing for multi-functional properties. PCL has been FDA approved for the design and use of sutures in surgeries, and therefore, its slow degradation and compatibility warrants use as a potential wound dressing [67]. Specifically, PCL fibers are used to treat wounds because the fibrous structure is similar to that of the ECM. PCL is especially advantageous because it has excellent water retention capacities that capture wound exudate [68]. Because of its lack of antimicrobial effects, PCL is often integrated with other polymers and/or antibiotics. Silver nanoparticles incorporated in a PCL matrix have shown inhibited bacterial invasion. One study also increased the hydrophilicity and diffusion properties by enriching PCL with nanochitosin (NC). NC provided antibacterial activity as well as sustained release of curcumin, a supplement used to reduce inflammation and pain, while providing better skin health and controlling cholesterol and blood sugar [69]. The combination of PCL and chitosan provided a tunable wound dressing for enhanced drug delivery and ultimately healing.

### 4.3. Smart Polymers

In recent decades, smart polymers have been heavily researched due to their important characteristics pertaining to thermal, chemical, and physical responses needed to modify the healing process. Unlike traditional synthetic or natural polymer wound dressings, smart polymers can control material properties in response to external cues [75]. The following studies highlight recent work in the analysis of smart wound dressings’ properties and the mechanism used to create responses.

A stimulus-responsive hydrogel is a soft, hydrophilic material that swells upon the absorption of water. They can be fabricated using copolymers, blends, or interpenetrating networks (IPNs) and made to respond to various changes in their environment, such as pH, temperature, chemicals, light, electric field, and shear stress [76]. Since pH values change overtime in chronic wounds [76], a pH responsible hydrogel can swell or contract when exposed to an overwhelming pH and ionic strength in the wound. Additionally, mechanical perturbations can be monitored by shear-responsive hydrogels. Hydrogels exhibit viscoelastic mechanical behavior upon deformation and will strain in a time-dependent manner upon application of stress, such as loading and pressure beneath the ulcer bed. Once an external stress is removed, the hydrogel can recover its original structure [76].

**Figure 3:**
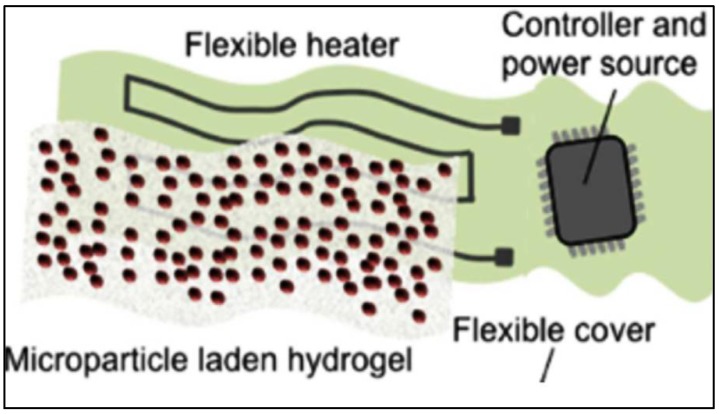
Schematic of the smart wound dressing depicting the major elements. Reprinted by permission from John Wiley and Sons: Advanced Healthcare Materials reference [78] Copyright 2016.

Dermal patches can be engineered by constructing a thermoresponsive drug microcarrier encapsulated within a hydrogel layer. *N*-Isopropylacrylamid (NIPAM) is a type of thermoresponsive material commonly used as a drug vehicle. The hydrophilicity of the material is dependent on temperature; it is hydrophilic below its critical temperature, 32 °C, and hydrophobic above. It can be tuned through copolymerization, allowing for optimal drug release [77]. Bagherifard et al., designed a smart, hydrogel-based, dermal patch with integrated heating elements for on-demand drug and growth factor delivery. The patch included a Ca-alginate hydrogel sheet, micropatterned gold heating elements, and the thermoresponsive NIPAM particles as seen in Figure 3 [78].

In addition to proper conformability with the skin, the ability to maintain skin moisture and protecting the wound from pathogens, the thermoresponsive drug carriers within the chosen hydrogel controlled for drug release [79].

Shape memory polyurethanes (SMPUs) are being incorporated into composite materials to provide more ideal mechanical properties and stretching capabilities [80]. The material has shape memory function that can control deformation and force, particularly in suture applications [81]. Tan et al., proposed to design a composite nanofibrous mat that consisted of chitosan, gelatin, and SMPU through electrospinning. The gelatin and chitosan contributed to wound healing as the incorporation obtained surface wettability, cytocompatibility, hemostatic properties, and water vapor transmission. Whereas, the SMPU allowed for controllability upon tensile force under different strains. In wound healing applications, the SMPU can eventually assist in the closure of cracked wounds [82].

An ideal smart wound dressing can impose the idea of an individualized healing function fit to a patient’s need, while responding to biological, chemical, and physical responses in the chronic wound environment. By doing such, drug delivery can be controlled in response to environmental cues and ultimately, prevent delayed healing.

### 4.4. Fiber Geometry and Scaffold Architecture

In addition to material type, geometry and conformation of the wound dressing can have just as big of an impact on successful healing. Important parameters to consider are porosity, dimension, and strength of the graft as well as macrostructure to ensure orderly extracellular matrix deposition. Typically, one material does not fit all criteria. We propose that a multilayered wound dressing is necessary to ensure all advantageous effects. A porous, hydrophobic contact layer is needed to create a barrier between the outside environment and wound, while promoting sterility and reepithelialization. A hydrogel or nonstick material is best to prevent further damage to the granulated tissue. A second and third layer is needed and relies on fibers oriented specifically to provide channels that can wick away wound exudate both vertically and horizontally. Fiber cross-section is an important variable to consider as it can alter capillary movement of fluid. In a previous study, permeability and fluid wicking were tested as a function of weave configuration and fiber geometry of various combinations of poly-l-lactide and poly-l-lactide-co-ε-caprolactone. The outcomes suggested that grooved wicking geometry, as opposed to a round cross-section, may be used in scaffold development to regulate fluid transport toward the area of interest [83].

A second parameter to consider is a single or multi-filament architecture as a component of the scaffold, especially for antibiotic delivery. A second experiment investigated the elution profile of gentamicin in a bundle of small wicking fibers and a single large fiber in order to test the effect of cross-sectional diameter on diffusion. The burst release of the large single fiber was substantially greater than the bundle of fibers, and the cumulative release was higher at each time point. Researchers concluded that the bundle had a slower release profile due to a reduced surface area in contact with the buffer solution when compared to the single large fiber. Although it has a slower release, the fiber bundle exhibits a stable and consistent elution profile for a larger duration.

Finally, fibers can orient themselves to form a specific type of porous fabric to allow tissue ingrowth and permeation of metabolites. Fabrics can be classified as woven, knitted, and non-woven, each having their own unique properties. Woven fabrics can be used where mechanical strength is required due to their low elongation, high breaking strength, and more mechanical stability. Knitted fabrics can provide good wrinkle and crush resistance, higher elongation, good elastic recovery and permeability, allowing for optimal tissue ingrowth. Wang et al., designed a PLGA knitted mesh-reinforced with collagen-chitosan scaffold and analyzed the geometry’s role in tissue regeneration. Researchers noted that the knitted-mesh provided mechanical strength, while inhibiting wound contraction and promoting neotissue formation and blood vessel ingrowth [70]. Non-woven fabrics are typically fabricated using techniques such as electrostatic or solution blow spinning. The fibers are staple-length and usually good for low strength applications. Electrospinning can create continuous fibers with diameters as small as a few nanometers, which possess a similar architecture to the structure of the ECM. Also, the porous feature of electrospun scaffolds allows for a high surface-to-volume ratio necessary for optimal cell attachment and oxygen and nutrient transport. Yang et al., discovered a higher wound recovery rate with complete skin regeneration. Electrospun fibrous mats were fabricated to allow gaseous and fluid exchanges, while absorbing exudate and wound odor. With the release of bFGF, enhanced collagen deposition and ECM remodeling was similar to normal tissue [84].

## 5. Biosensing in the Chronic Wound Environment

In addition to smart, adaptive wound dressings, researchers and clinicians have recently realized the need for quantitative assessment of the chronic wound environment through biosensing. There is a need for a smart dressing for chronic wounds that is composed of sensing elements that can: (1) be integrated with current biological dressings; (2) sense changes at the wound microenvironment; (3) provide data to the health care professional; and (4) serve as a theragnostic approach by releasing antimicrobials when needed [85,86]. This need stems from current assessment procedures (Table 1), which are mainly subjective and require a healthcare professional to analyze and classify wounds without any readily quantifiable data [87]. Much of the work in biosensing of chronic wounds is focused on the ability to discern the probability of wound closure and finding the quantitative thresholds of various biomarkers related to wound healing [11]. A summary of these developments in sensing the chronic wound environment will be discussed in the following sections.

### 5.1. Biomarkers for Wound Healing

Several biomarkers in chronic, non-healing wounds have been identified as potential indicators for the management and treatment of chronic wounds. These biomarkers can be divided into two major categories, (1) biochemical and (2) physical. Each can be quantified through electrochemical or electrophysical transduction, where some biochemical marker (i.e., cytokine, enzyme, metabolic byproduct, pH, etc.) or some physical marker (i.e., temperature, pressure, moisture level, etc.) is converted to a measurable electrical signal. Researchers have also explored different methods for qualitative measures and visualization of wound healing. Various spectroscopy methods, such as absorption, fluorescence, phosphorescence, Raman, SERS, refraction, and dispersion spectrometry are used to detect changes in energy, polarization, amplitude, decay time, and/or phase. Amplitude analysis is one of the most widely used methods for correlation of analyte concentration with signal amplitude [88].

#### 5.1.1. Biochemical Markers

There are many metabolic pathways in the wound healing process and further research is needed to identify key biomarkers and their respective roles in each pathway. Cytokines, proteases, bacteria, oxygen, nitric oxide (NO), etc., are potential biochemical markers that could predict non-healing wounds [11,89]. Cytokines are cell signaling molecules secreted by platelets, fibroblasts and inflammatory cells during inflammation. IL-1, IL-6, and TNF-α are cytokines that have been measured in higher concentrations in chronic non-healing wounds when compared to normal healing wounds [11]. Beila and coworkers have demonstrated that the overall pro-inflammatory level of 16 tested cytokines were higher in ulcer tissues than normal tissues before treatment of chronic venous insufficiency ulcers [90]. Proteases are the enzymes responsible for breaking down proteins. Protease levels have also been shown to be higher in non-healing wounds as compared to normal or acute healing wounds [11]. The major proteases of interest are MMP-2 and -7. MMPs, are involved in the inflammatory stage of wound healing and are released by inflammatory and connective tissue cells. They are responsible for breaking down necrotic tissue and the extracellular matrix prior to the proliferation stage of the normal wound healing cycle. Utz et al., have demonstrated with high probability (*p* < 0.001) that MMP-2 and -7 levels were significantly higher in chronic wounds than in acute wounds [91]. Bacteria concentration levels are abnormally higher in chronic wounds due to the fact that the wound is stuck in the inflammatory stage. Some of the most commonly found bacteria in chronic wounds are *S. aureus*, *E. coli*, and *P. aeruginosa* [92]. NO has been well established as a biomarker in wound healing and is known to have lower concentrations in chronic non-healing wounds [89]. However, NO has a half-life less than 10 s making it difficult to measure. Investigators have tried to measure wound fluid NO levels indirectly through wound fluid nitrate, induced NO response and fasting urine [89,93]. Boykin concluded that deprivation of NO activity contributes to impaired healing, and a comprehensive method to monitor NO, MMP, and bacterial load could accelerate healing in chronic wounds [89]. Wound pH and uric acid play a vital role in cell to cell interaction in wound healing. Change in pH is a good indicator of wound healing whereas a sudden alkalotic pH followed by a gradual decrease in pH to a steady-state value around 5–6 indicates proper wound healing [86]. UA concentration tends to be decreased in chronic wounds due to the increase of infection and bacterial consumption of UA [94]. The fabrication of biochemical sensors for some or all of these biomarkers would exploit a specific method of transduction, therefore enabling quantification.

#### 5.1.2. Physical Biomarkers

Physical biomarkers that have been explored for chronic wound healing are bioimpedance, pressure, and ambient temperature. Bioimpedance includes measurement of resistance, reactance and the associated phase angle. Resistance is related to the amount of extracellular fluid (ECF) in a given sample of tissue; reactance is related to the cell mass and is a good indicator of cell accumulation and proliferation; and phase angle corresponds to the vitality of the tissue and is a good prognosis of tissue nutrition [87]. The resistance to current arises from the fact that less ECF decreases the number of ions and lowers the conductivity of the matter. While the reactance value arises from the capacitive nature of cell membranes, lower values at a specified frequency value indicate decreased wound mass. Increased pressure hinders proper healing in foot ulcers; relieving pressure before the onset of an ulcer may prevent its genesis [95]. Increase in temperature is associated with increased bacteria levels, which often indicates complications in wound healing [96]. Table 3 lists the biomarkers that are associated with chronic non-healing wounds.

### 5.2. Biochemical Sensors

Biochemical sensors are designed for a specific analyte which makes them highly selective and sensitive. Ideally, a biosensor has a biorecognition element where the specified analyte binds to a transducer to change the chemical reaction or physical signal into an electrical signal, and a signal amplification and processing component [88]. These sensors are very complex, but very specific and promising in sensing the ulcer environment. The use of a sensor that could detect one of the biochemical markers listed in Table 3 above would be useful where the identification of a specific wound condition is needed. Table 4 summarizes the sensors discussed in this section.

#### 5.2.1. Matrix Metalloproteinases

MMP sensors require the use of enzymes that are capable of catalyzing a specific biochemical reaction under a desired condition. Signal reduction from bio-fouling is a major concern as unwanted biological molecules interfere with the reaction [97]. One of the most widely used types of protein assays is the enzyme-linked immunosorbent assay (ELISA). The ELISA consists of the immobilization of an antigen on a substrate, addition of a buffer solution, detection of a target analyte, and observation of the optical density via absorbance spectroscopy [98]. Milne and colleagues created a device that was able to detect wound pH, moisture content and the MMP activity [99]. The investigators created an ELISA sandwich assay to electrochemically detect the MMP activity in the wound site where they were able to detect MMP-9 concentrations between 0.1–100 ng/mL [99]. Biela et al., were able to make a disposable MMP-9 sensor that relied on the degradation of a peptide cross-linker [90]. The team used a microfabrication technique in order to fabricate 1.5 mm interdigitated gold electrodes with 0.1 mm thick gate channel [90]. Their design models a standard semiconductor field effect transistor. They used electrochemical impedance spectroscopy to analyze the presence of MMP-9 where they were able to detect 200 ng/mL of MMP-9 within 5 min [90].

#### 5.2.2. Uric Acid

The sensing of UA is a promising methodology that uses enzymatic sensing techniques. Kassal and colleagues designed a wireless UA sensor by the use of screen printing and immobilization of uricase on a working electrode with a −0.3 V operating voltage [94]. The sensitivity coefficient of the sensor to 100–800 uM of UA was 2.4 nA/uM UA with an on board potentiostat compared to an electrochemical analyzer [94]. The sensor remained highly selective for UA when compared to a control 400 μM UA solution. In the presence of common biological interferences and ascorbic acid, the values of UA concentration decreased by 3% and 10%, respectively [94]. Carbon fiber-based sensor electrodes have been developed by Sharp et al., to detect uric acid levels electrochemically. The electrodes were modified with the application of a cellulose acetate permselective barrier to avoid biofouling. The team reported a sensitivity range of 0–500 µM for a linear fit (R^2^ = 0.97) [100]. Choudhury and coworkers developed an enzymatic electrochemical sensor to detect uric acid changes in real-time by the immobilization of uricase and detecting the byproduct H_2_O_2_. The team was able to achieve a sensitivity of 0.14 µ/M-cm^2^ and a limit of detection of 14 µM [101].

#### 5.2.3. pH

Most pH sensors utilize fluorescent or colorimetric agents to measure the pH level of solutions. This could be achieved by inserting a pH sensitive dye into the fiber matrix of a desired dressing. The pH range of the sensor depends on the receptor type where a hydroxyl groups or an amine group are the main receptors of choice [102]. McLister and Davis demonstrated the use of a poly-tryptophan modified carbon fiber composite to detect wound pH [103]. They utilized square wave voltammetry to detect the pH of electrogenerated indolic quinone moieties and horse blood. The voltammograms varied with pH and were fit to a linear model over a range of pH from 3 to 8 (R^2^ = 0.993) [103]. Tamayol et al., fabricated a composite smart sensor that utilized alginate-based fibers that responded to changes in pH by changing colors. Microfluidic spinning method was used to fabricate alginate and glycerol solution with mesoporous particle beads into fibers. Fabricated fibers were placed in a PDMS chamber that received an inlet and outlet of solutions of varying pH of 6.5 to 8. The fibers changed color to dark red with basic and yellow with acidic solutions [79]. Real-time sensing was achieved with the use of a smartphone camera and a custom MATLAB code to detect the pH of pig skin samples with the use of RGB values. The sensor values were within ±0.2 pH units compared to actual values [79]. A hydrogel-based inductive pH sensor was used to detect pH changes in chronic wounds [104]. The coils of the sensor were placed on the top and bottom surface of a folded substrate and a poly(vinyl alcohol)-poly(acrylic acid) pH-sensitive hydrogel was sandwiched between the substrate surface. The coils’ mutual inductance depended on the gap created by the hydrogel. The sensor displayed a linear response to pH in various buffer solutions between 1 to 8 [104].

#### 5.2.4. Bacterial

Several sensors that detect the presence of bacteria have been developed by researchers using many of the same principles previously discussed. A team from the University of Rochester Medical Center fabricated a device using electrochemical multilayered porous silicon fibers [105,106]. Lipid A was one of the target molecules and Tertryptophan ter-cyclopentane (TWTCP) was used as a receptor to bind to the target molecule, diphosphoryl lipid A [105,106]. This binding created a change in the refractive index of the silicon that created an 8 nm shift in the wavelength of its photoluminescence peak. Gram-positive bacteria induced no change, but gram-negative bacteria produced a small wavelength shift of 3–4 nm in the presence of bacteria. DeLouise tested the sensor on *E. coli* but failed to detect any changes due to a high rate of false negatives because of the pore blocking preventing target infiltration [107] A similar technique was used by Thet et al., where they demonstrated the use of colorimetric detection to identify the presence of bacteria. Hydrated agarose film containing 5,6-Carboxyfluorescein dye vesicles were mixed with the agarose film. Compared to a 4-(2-hydroxyethyl)-1-piperazineethanesulfonic acid (HEPES) control group, detection of *S. aureus* and *P. aeruginosa* was seen with an intensity contrast approximately 20,000 and 35,000 fluorescence/a.u. after 24 h. However, they were unable to detect any changes in *E. coli* or *E. faecalis* culture [107].

#### 5.2.5. Nitric Oxide

**Figure 4:**
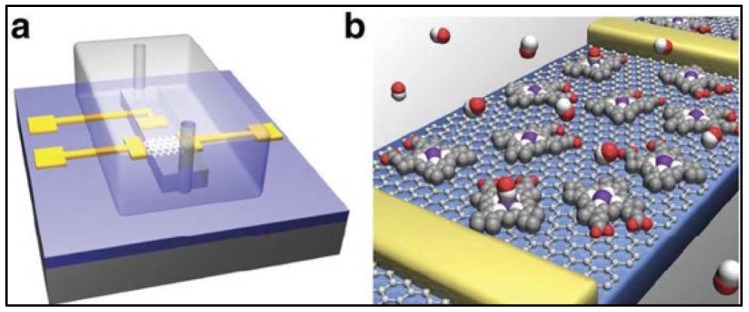
NO hemin-functionalized sensor. (**a**) PDMS channel with gold gate, source, and drain electrodes; (**b**) NO selective channel. Reprinted by permission from Springer Nature: Nature Communications reference [108]. Copyright 2013.

NO sensors measure the concentration of NO in a solution by the electroreduction or electrooxidation of NO. This could be accomplished by the utilization of a working electrode that has a potential of −0.5 to −1.4 V (electroreduction) and 0.6 to 0.9 V (electrooxidation) vs. AG/AgCl [93]. However, these electrodes experience biological interferences such as L-arginine, sodium nitrate, sodium nitrite, oxygen, and hydrogen peroxide [89,93]. Biological interference is filtered out by the inclusion of a transition metal or a metalloprotein such as hemoglobin [93]. Most common sensors are of the Shibuki-style, solid permselective, and solid catalytic [88,93]. The mentioned sensors are hard to utilize within a dressing and a custom sensor would be needed for application in chronic wounds. A novel method to electrically detect nitric oxide at subnanomolar levels was found by the use of a hemin-functionalized graphene field effect transistor (FET) [108]. The device consisted of titanium-gold source, drain, and a solution gate. A PDMS channel was added to test the presence of NO in NONOate sodium in a NaOH solution. Presence of NONOate sodium at the device active site caused the conductance to gradually decrease from 160 µS to 135 µS with a half-life of 135 s [108]. Figure 4 is an illustration of the Hemin-functionalized graphene FET. Furthermore, the sensor was able to detect as low as 0.3 nM of NO with a signal-to-noise ratio(SNR) of 3 s [108].

#### 5.2.6. Oxygen

Oxygen sensors work similarly to NO sensors where the produced current is proportional to the given concentration of the solution. The Clark oxygen amperometric sensor is the most common type of oxygen biosensor [88]. A wireless oxygen bandage sensor was fabricated by the use of a 3D printed polyrene-C dressing and the use of commercialized electronic components [109]. Mostafalu and coworkers used a galvanic cell of 0.8 V that was said to be proportional to the reduced oxygen at the cathode and oxidized zinc at the anode. Ag and Zn electrodes were submerged in a potassium hydroxide electrolyte and capped off by a thin oxygen permeable PDMS top layer. The current from the oxygen sensor was amplified to 1–5 V by an analog front-end, then read by a microcontroller with a 1 Hz sampling frequency. The signal was sent wirelessly to a monitor for data visualization and analysis. Current output from the sensor was 5–700 µA with an internal gain between 2.75–350 K ohms [109]. The ability to get such a wide range of output current and gain allows the sensor to be able to detect oxygen at different concentrations. The investigators reported current between 0.4–0.6 mA as oxygen concentration changed over 200–450 s [109].

### 5.3. Physical Sensors

Physical sensors incorporate the use of an analog-to-digital converter, amplifier and signal processing component to produce quantifiable data. Capacitive, resistive, and thermoelectric sensors are examples of physical sensors used in biomedicine. Physical sensors measure a parameter based on emergent phenomena or a cascade of metabolic activities. Thus, physical sensors are not as specific as biochemical sensors. However, they are widely used because of their ease of use and robustness.

#### 5.3.1. Impedance

Impedance sensors measure the resistance, reactance, and phase angle associated with the wound location by applying a small non-perturbing current and detecting the voltage that is produced. Due to the fact that chronic wounds have a higher level of bacteria, the conductance of the wound tends to be higher [94]. Farrow et al. reported the development of a real-time impedance sensor that was able to detect the presence of *S. aureus* cultures by analyzing the normalized impedance profiles with respect to the frequency spectrum [110]. The researchers developed a screen-printed sensor that contained Ag/AgCl electrodes inside of a 30 mL bacterial test vial [110]. They were able to detect *S. aureus* strains at levels of 5 × 10^7^ CFU/mL [110]. They also showed that impedance measurements inhibited the growth of bacteria, due to the deposit of Ag ions in the culture [110]. Ag is a widely acceptable antimicrobial molecule and the use of Ag in wound dressings has been demonstrated clinically [23].

**Figure 5:**
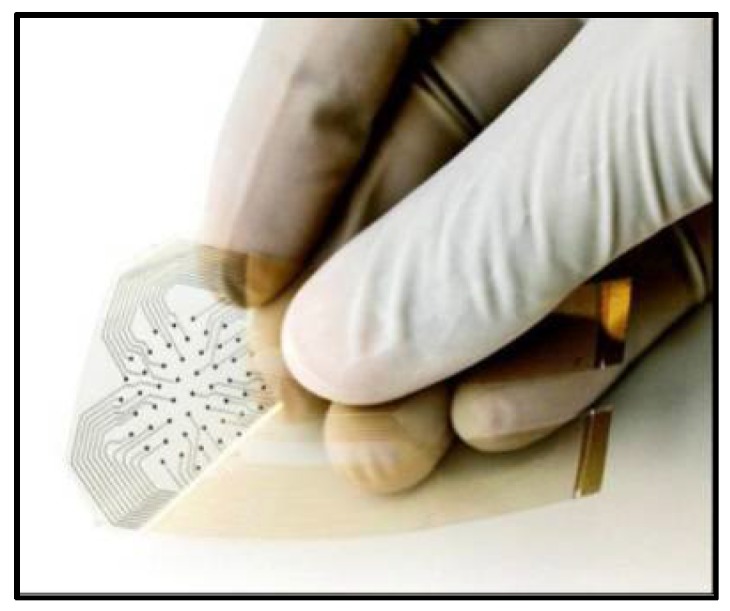
Flexible impedance sensor made via Inkjet Printing. Reprinted with permission from Springer Nature: Nature Communications reference [115] Copyright 2015.

Swisher et al. designed a flexible, electronic device that non-invasively detects ulcers via impedance spectroscopy across a flexible electrode array in a rat model [115]. They used inkjet printing to create gold-plated electrodes on a polyethylene naphthalene substrate where they used a LCR meter to detect the impedance between two locations on the array. The measurements were gathered in a scanning fashion, covering the entire sensor [115]. This method effectively captures more surface area than a single two electrode measurement. The researchers compared the data from the LCR meter to a histology graph and showed that at 15 kHz the threshold for tissue damage was seen when the impedance magnitude was 6 K ohms and the phase angle was between −30 and 10 degrees [115]. Figure 5 shows an image of the hydrogel impedance array sensor used for the study.

#### 5.3.2. Temperature Sensors

The most commonly used temperature sensors are thermistors, thermocouples, resistive temperature detectors (RTDs), and digital/analog (AD) thermometers. Thermistors and RTDs have a resistive element that has a negative non-linear relationship to the given temperature. RTDs also have a resistance-temperature relationship but are typically more sensitive. Thermocouples utilize two metal probes to detect a change in temperature by sensing a potential difference between the probes. AD thermometers utilize integrated circuits to capture a temperature value. A thin, flexible micro-fabricated platinum resistor temperature sensor was fabricated by Kim and colleagues [111]. Resistance-temperature curve shows that the sensor is linear for temperature in the range of 0 °C to 120 °C with a slope of 2.7 Ω/°C. Matzeu et al., developed a wireless thermistor sensor based on resistive carbon nanotube film and radio frequency identification (RFID) tag [112]. The team did not find a linear relationship between temperature and resistance. At 35 °C they obtained a slope of 17 Ω/°C for their resistance-temperature curve. The sensor’s measurement resolution was reported to be 0.2 °C [112].

#### 5.3.3. Integrated Sensors

Some sensors use a collection of modalities that are integrated on a single chip. Pressure, moisture, pH, irregular bleeding, and/or temperature are integrated on a single device in order to capture a holistic view of the wound. Mehmood et al., used a wireless sensor to inspect the wound’s moisture level, temperature and pressure [113]. They used commercially available devices such as the Honeywell HCZ-D5, Interlink Electronics FSR406, and Texas Instrument LM94021B for the moisture, temperature and pressure sensing, respectively. Under standard conditions and room temperature, they were able to detect a temperature difference of 0.2 °C. They also detected very low resolution for pressure and moisture of 0.5 mmHg and 3.0% RH, respectively. These values are sensitive enough to detect a change in the chronic wound environment. In addition to sensing, a telemetry system was incorporated with the use of the ZigBee network protocol [113]. ZigBee was used due to its ease of use for experimental verification of their device. However, a Wi-fi Protected Access II (WPA2) protocol would be more ideal due to its inherent security and its use in the hospital settings [113]. Farooqui and Shamim fabricated a disposable bandage via inkjet printing that used a double layer technique to measure the irregular bleeding, pH, and external pressure of a wound with wireless telemetry capabilities. This was achieved by detecting any significant changes to the initial capacitance and resistance [114]. They were able to correlate the change in resistance, dielectric properties of blood and the change in distance to pH level, bleeding and pressure, respectively [114]. Similar sensors were made that employed pressure sensors inside of shoes, such as the insole flexor force sensor used by Ostadabbas et al. [116].

## 6. Sensor Clinical Outcomes

Direct clinical applications of biochemical sensors to chronic wounds are discussed here, although the authors found reports of such studies to be limited. A team from the University of Swansea will begin a clinical trial that will utilize a sensor capable of detecting coagulation and/or infection [117]. Another clinical trial examined the NO levels of ten human vastus lateralis muscle biopsies by the use of an assay and a solid-state gas amperometric sensor. The team used an amperometric solid-state sensor to measure the pmol/mg of NO in muscle protein and fresh tissue. NO levels increased to approximately 50 pmol/mg of protein, then attenuated to 45 pmol/mg of protein after 20 min of reperfusion [118]. This study illuminates the potential of sensing NO levels in chronic wounds. Schreml et al., used a sprayable luminescent pH sensor to examine the wound status in vivo [119]. Fluorescein isothiocyanate and aminoethyl cellulose (FITC-AC) were covalently linked to form pH-sensitive particles [119] RGB values were found from the pH-dependent green (G) channel and the pH-independent red (R) channel reference signal. They found the referenced pH signal for each pixel in the data from the G/R ratio. In a split skin donor site, pH decreased prior to wound closure [119].

Physical sensors have been extensively used in clinical trials to quantify the wound healing process. Liao et al., applied a modified hydrogel-based impedance sensor to a clinical study monitoring five patients with stage I, II and III ulcers [116,120]. They found the impedance threshold for tissue damage to be higher than data from a previously studied rat model at 100 K ohms in magnitude and a phase angle between −30 and 10 degrees [116,120]. Milne et al., recruited 30 patients for their observational clinical trial where they used a commercially available moisture sensor (WoundSense) that uses impedance to measure the degree of moisture in wounds at time of dressing change [121]. The wounds were identified as either wet, wet to moist, moist to dry and dry [121]. DFUs were found to have a higher moisture content than pressure ulcers. Out of the 30 patients examined they found that 44.9% of the 588 dressings were in optimal condition prior to changing [121]. The significance was their ability to show that wound dressings were changed too frequently by healthcare professionals and the use of their real-time monitoring system would be economical and more efficient for the wound healing process.

Researchers have investigated methods to quantify the healing process via bioimpedance. Lukaski and coworkers conducted an experiment where they measured the impedance of a wound [87]. They used a controlled electrical circuit that included the wound and four small adhesive electrodes placed on the outer bounds of the wound site. They applied a 50 kHz low-voltage to the wound and generated a small current in order to measure the impedance. They found that after treatment of an acute wound the resistance increased until epithelialization, reactance increased, and phase angle increased as well [87]. However, after a graft on a non-healing wound, the resistance, reactance and phase angle decreased by 2%, 28%, and 30%, respectively [87]. Moreover, MRSA infected wounds generated a significant decrease in resistance, reactance and phase angle by 16%, 63%, and 56%, respectively [87]. The investigators note that cell membrane disruption and function generated the decrease in impedance values, which also validates the accepted theory that impedance decreases as wound degenerates. Kekonen et al., conducted a similar study, but instead calculated the healing ratio as a percentage value of the overall wound impedance of a single person [122].

A multifunctional skin-like sensor was design by Hattori et al., the composite smart dressing used a multilayer construct that consisted of metal traces with fractal geometries. Temperature sensors were integrated in the device via transfer printing, coating and encapsulation of a silicone layer. Temperature was detected by the use of an IR camera. The multilayered sensor was conformal to all three wounds and was able to detect a temperature rise in all three subjects with the assistance of an IR camera [123].

## 7. Conclusions

There is a limited number of options for successfully treating diabetic chronic wounds and the need to discover a solution that considers all parameters involving inflammation and repair is vital. A smart dressing fabricated from optimal material combinations oriented in a specific multi-layered architecture needs to possess qualities that inhibit bacterial growth, manage excess exudate, and promote re-epithelialization, all while maintaining a moderately moist environment. Composites of natural and synthetic polymers can contribute a similar composition as ECM in addition to delivering antibiotics and growth factors. Fiber geometry and scaffold type can also affect antibiotic elution, mechanical strength, and porous structure for tissue growth. These wound smart dressings can be designed to address clinical problems, but alone, they cannot identify the particular state of the wound.

Biosensing offers a unique set of quantitative advantages in real time, such as exudate levels, bacteria concentrations, and tissue regeneration. There are many sensor types and potential target biomarkers. However, for them to be useful in sensing the wound environment they need to be disposable, biocompatible, able to detect clinically relevant parameters, conform to the shape of the ulcer and have flexible properties similar to the applied dressing. Biochemical sensors provide the necessary sensitivity, but miniaturization and integration with a smart dressing would be more difficult than the use of an impedance or pressure sensor device. The use of a biochemical sensor would provide increased specificity, but these devices are typically limited with respect to shapes for fabrication. In contrast, impedance and pressure sensors could be fabricated as an array that could be implanted into the dressing of choice. Further research and investigation needs to be done to assess the use of biological, impedance, or pressure sensors in the use of real-time detection of the chronic wound environment. Advancement in the integration of a smart dressing would help prevent ulcers and amputation and accelerate the healing process. Sensing the chronic wound environment in real-time and creating a feedback system would quantify and classify the healing process and equip clinicians with a valuable tool to quickly identify the degeneration of a chronic wound.

## Figures and Tables

**Table 1:** Major classification methods.

	Wagner-Meggitt	University of Texas	PEDIS
**Grade 0**	Pain only, no open ulcer	Pre-ulceration	
**Grade 1**	Superficial ulcer	Superficial wound	Skin intact, no infection or loss of sensation
**Grade 2**	Deep ulcer	Wound penetrating to tendon or capsule	Superficial ulcer with infection at the surface and loss of sensation
**Grade 3**	Deep ulceration with osteomyelitis	Wound penetrating to bone or joint	Ulcer reaching the fascia, muscle, and tendon, fasciitis and septic arthritis likely
**Grade 4**	Localized Gangrene		Ulcer depth reaching the bone or joint, SIRS
**Grade 5**	Extensive Gangrene, Amputation likely		
**References**	[13,16,17]	[18]	[19]

**Table 2:** Summary of Polymer-based Wound Dressings.

Polymer	Advantages	Disadvantages	Reference
Cellulose	1. Readily available with low cost 2. Fiber and foam materials 3. Creates a gel-like material, forming a moist environment 4. Releases GFs to stimulate fibroblast proliferation	1. Requires additional antimicrobial substances 2. Resorption in tissues does not occur, which could cause further tissue damage or become overwhelmed by excess exudate	[36]
Chitosan	1. Fabricated in a gelatin of film-like material 2. Antimicrobial and hemostatic properties 3. Functional derivatives allowing for modified and versatile effects 4. Ability to deliver drugs	1. Extensive swelling in water 2. Unable to dissolve in organic solvents because of its rigid crystalline structure	[38,43,44,45,70,71]
Collagen and Gelatin	1. Promotes tissue granulation and angiogenesis 2. Inhibits bacterial growth and prolonged inflammatory response 3. Gelatin derivative forming a hydrogel material	1. May not be absorptive in gelatin form, especially for wounds with excessive exudate 2. Might require secondary dressing	[22,38,46,48]
Hyaluronic Acid	1. Lubricative and water absorptive 2. Bi-products promote epithelial cell migration 3. Improves collagen deposition and angiogenesis4. Popular drug delivery system and vehicle for growth factors	1. Only MMWHA enhances wound repair	[52,72,73,74]
Poly(lactide-co-glycolide)	1. FDA approved for drug delivery, suture applications 2. Ratio of lactide to glycolide units can modify release of drugs and growth factors 3. Cytocompatible and stimulates fibroblast adhesion, spreading, and proliferation 4. Fabricated into various shapes	1. Requires additional antimicrobial substances 2. Properties fail to match ECM or collagen	[38,56,57,58,59]
Polyurethanes	1. Semipermeable membrane that prevents bacteria from entering 2. Provides a moist environment 3. Delivers bioactive substances for fighting infection 4. Drainage properties that decrease the risk of swelling	1. Need composite dressings in order to provide contact layer and waterproof properties 2. Wound healing effects are only associated with nanofiber structure	[57,61,62]
Poly(ethylene glycol)	1. Hydrophilic, flexible and compatible qualities 2. Surface modifier allowing for better grip in the contact layer 3. Growth factors have higher affinity for PEG	1. Adhesiveness might damage granulation tissue 2. Does not incorporate antibiotics and other drugs so composite materials are needed	[38,57,64]
Polycaprolactone	1. FDA approved for suture applications 2. Fibrous structure similar to ECM architecture 3. Water retention capacities used to capture wound exudate 4. Resistant to many solvents allowing for slow and controlled degradation	1. Lack of antimicrobial properties	[67,68,69]

**Table 3:** Biomarkers associated with chronic wound healing.

**Biochemical Biomarkers**		
**Wound Biomarker**	**Significance in Chronic Wounds vs. Acute Wounds**	**Reference**
Cytokines (IL-1, IL-6, TNF-α)	Elevated levels of Cytokine	[11,90]
Nitric Oxide	Decreased levels of NO	[89,93]
Matrix Metalloproteinase	Increased protease activity	[11,91]
Oxygen	Higher probability for ischemia due to decreased oxygen levels	[9]
Bacteria	Bacteria concentration levels are higher indicating extent of infection.	[9,92]
Wound pH	Remains more alkalotic for extended period of time	[86]
Uric Acid	Decreased levels due to bacteria	[94]
Reactive Oxygen Species	Increased levels due to oxidative stress	[11]
Gene Expression	Increase in bacterial housekeeping genes; decrease in ulcer housekeeping genes.	[11]
Growth Factors	Decreased level (i.e., PDGF)	[11]
**Physical Biomarkers**		
**Wound Biomarker**	**Significance in Chronic Wounds vs. Acute Wounds**	**Reference**
Bioelectrical Impedance	Phase angle, resistance, and reactance are all decreased	[87]
Pressure	Increased pressure	[95]
Temperature	Increased temperature	[96]

**Table 4:** Sensor Used to Detect Biomarkers in Chronic Wounds.

Sensor	Sensitivity/Range	Biomarker	Method	Reference
ELISA MMP Sensor	0.1–100 mg/mL	MMP-9	Electrochemical	[99]
Disposable MMP-9 Sensor	200 mg/mL	MMP-9	Electrochemical Impedance Spectroscopy	[90]
Smart Bandage UA Sensor	100 µM of UA	Uric Acid	Electrochemical	[94]
Carbon fiber sensor	0–500 µM	Uric Acid	Electrochemical	[100]
Wearable enzymatic sensor	0.14 µ/M-cm^2^ Range: 14 µM	Uric Acid	Electrochemical	[101]
Poly-tryptophan Carbon Fiber pH Sensor	pH of 3–8 (±0.1)	pH	Voltammetry	[103]
Flexible Hydrogel pH sensor	pH of 5–8 (±0.2)	pH	Fluorescent Spectroscopy/Image processing	[79]
Hydrogel pH sensor	pH of 1–8	pH	Electrical (LC circuit) and Chemical	[104]
Smart Bandage	Gram-Negative Bacteria, shift in wavelength by 3–4 nm	Gram-negative,-positive, *E. coli*, Lipid A	Electrochemical/Optical Microcavity	[105,106]
Intelligent Hydrogel Dressing	Contrast of approximately 20,000 and 35,000 fluorescence/a.u. of *S. aureus* and *P aeruginosa*, respectively compared to HEPES	Bacteria (*S. aureus*, *P. aeruginosa*, *E. coli*, *E. faecalis*)	Electrochemical/Fluorescent Spectroscopy	[107]
Hemin-Functionalized FET NO Sensor	0.3 nm of NO	Nitric Oxide	Bio-electrical	[108]
Oxygen Bandage Sensor	0.4–0.6 mA	Oxygen	Bio-electrical	[109]
Screen Printed Impedance Sensor	5 × 10^7^ CFU/mL of *S. aureus*	*S. aureus*	Electrical	[110]
Flexible Pt thermistor	2.7 Ω/°C	Temperature	Electrical	[111]
Wireless thermistor	17 Ω/°C at 35 °C	Temperature	Electrical	[112]
Flexible Low Power Sensor	0.2 °C temperature difference, 0.5 mmHg pressure, 3.0% RH	Moisture, Temperature, Pressure	Electrical	[113]
Inkjet Printed Smart Bandage	±2.3% capacitance, 8% quality factor, ±2.6% resistance	Blood, pH, Resistance	Electrical (Capacitance and Resistance)	[114]
Flexible Sensor Array	100–50 KΩ at 100–1 MHz	Impedance	Electrical	[115]
